# Mini-HA Is Superior to Full Length Hemagglutinin Immunization in Inducing Stem-Specific Antibodies and Protection Against Group 1 Influenza Virus Challenges in Mice

**DOI:** 10.3389/fimmu.2018.02350

**Published:** 2018-10-12

**Authors:** Joan E. M. van der Lubbe, Johan W. A. Verspuij, Jeroen Huizingh, Sonja P. R. Schmit-Tillemans, Jeroen T. B. M. Tolboom, Liesbeth E. H. A. Dekking, Ted Kwaks, Börries Brandenburg, Wim Meijberg, Roland C. Zahn, Ramon Roozendaal, Harmjan Kuipers

**Affiliations:** Janssen Vaccines and Prevention, Pharmaceutical Companies of Johnson and Johnson, Leiden, Netherlands

**Keywords:** universal influenza vaccine, hemagglutinin stem immunogen, influenza, mouse influenza challenge models, antibodies

## Abstract

Seasonal influenza vaccines are updated almost annually to match the antigenic drift in influenza hemagglutinin (HA) surface glycoprotein. A new HA stem-based antigen, the so-called “mini-HA,” was recently shown to induce cross-protective antibodies. However, cross-reactive antibodies targeting the HA stem can also be found in mice and humans after administration of seasonal vaccine. This has raised the question whether in similar conditions such a mini-HA would be able to show an increased breadth of protection over immunization with full length (FL) HA. We show in mice that in a direct comparison to H1 FL HA, using the same immunization regimen, dosing and adjuvant, a group 1 mini-HA has a higher protective efficacy against group 1 influenza virus challenges not homologous to the H1 FL HA. Although both antigens induce a similar breadth of HA subtype binding, mini-HA immunization induces significantly more HA stem-specific antibodies correlating with survival. In addition, both mini-HA and H1 FL HA immunization induce influenza neutralizing antibodies while mini-HA induces significantly higher levels of mFcγRIII activation, involved in Fc-mediated antibody effector functions. In agreement with previous findings, this confirms that more than one mechanism contributes to protection against influenza. Together our results further warrant the development of a universal influenza vaccine based on the HA stem region.

## Introduction

Influenza is a major global health problem causing serious morbidity, mortality, and substantial productivity loss each year. The most cost-effective strategy to prevent influenza is by vaccination (1, 2). Although current seasonal vaccines are often effective, a mismatch between circulating virus strains and the strains included in the vaccine occasionally reduces vaccine efficacy (3). In addition, seasonal vaccines have limited effectiveness against influenza strains newly introduced in the human population (4).

The discovery of influenza HA-specific antibodies in humans which are able to neutralize a broad spectrum of influenza A and B strains (5) has raised hope for a cross-protective influenza vaccine able to elicit these broadly protective antibodies. Such a universal influenza vaccine could mitigate the problems of mismatch between the vaccine and circulating strains, as well as providing protection to novel pandemic influenza strains (6). Interestingly, cross-reactive antibodies can also transiently be induced in mice and humans by one or multiple immunizations with a seasonal vaccine (7–9). Induction of these antibodies in humans correlated with protection of mice against lethal challenge with a genetically distant H5N1 influenza strain after passive antibody transfer (10). However, the majority of antibodies elicited in humans by vaccination or exposure to influenza A bind to the immunodominant and highly variable hemagglutinin (HA) head epitopes, often specific for one virus strain (11–13). In contrast, most influenza A cross-reactive broadly neutralizing antibodies (bnAb) target the highly conserved HA stem region, but are present at a relatively low frequency in humans (14–16).

Various approaches to induce cross-protective antibodies targeting the less immunogenic stem region were tested such as sequential immunizations with chimeric HA molecules, composed of different HA head regions on the same stem region (17–19), and shielding of the HA head epitopes by hyperglycosylation (20, 21). Another strategy directing the immune response to the more conserved HA stem is by removing the immunodominant head region, constructing headless HA stem-based antigens. We recently described a stable trimeric headless group 1 influenza HA, the so-called “mini-HA,” while others pursued a hemagglutinin-stem nanoparticle based approach; both approaches led to formulations that were immunogenic and cross-protective against different phylogenetically distant influenza virus strains in mice, non-human primates (NHP) and ferrets (22–26).

However, the epitopes recognized by cross-reactive antibodies are also present in full length HA and cross-protective immune responses can be found in humans and mice after multiple immunizations with a seasonal vaccine (7–9). This has raised the question how the immune response to mini-HA compares to the response to H1 full length (FL) HA as found in seasonal vaccines.

In this study we directly compared immunization with H1 FL HA A/Brisbane/59/07 (H1 FL HA) to a group 1 mini-HA which consists of the same backbone (23), using equimolar doses, the same immunization regimen and adjuvant in different influenza challenge mouse models. We show that immunization with mini-HA induces significantly better protection against lethal H1N1 and H5N1 challenge compared to H1 FL HA. Although both antigens induce a similar breadth of HA subtype binding, mini-HA immunization induces significantly more HA stem-specific antibodies correlating with survival. In addition, both mini-HA and H1 FL HA immunization induced influenza neutralizing antibodies while mini-HA induced significantly higher levels of mFcγRIII activation, involved in Fc-mediated antibody effector functions.

## Results

### Immunization with mini-HA induces cross-protection against H5N1 and H1N1, in contrast to immunization with H1 FL HA A/brisbane/59/07

Mice received three intramuscular injections with a dose range of either trimeric mini-HA [UFV4900, also known as #4900 (23)], or equimolar amounts of trimeric H1 FL HA A/Brisbane/59/2007, 3 weeks apart. Using differential scanning fluorimetry, the mini-HA starts to unfold around 52°C, and H1 FL HA around 50°C, suggesting the thermal stability of the antigens to be similar. Negative controls were mock immunized with Phosphate buffered Saline (PBS). All immunizations were adjuvanted with aluminum hydroxide, Al(OH)_3_, hereafter referred to as Alum. Four weeks after the third immunization mice were challenged with 12.5xLD_50_ of influenza virus. To compare the cross-protective efficacy of mini-HA immunization to H1 FL HA we selected two influenza strains with genetically distant HAs; H1N1 A/PuertoRico/8/1934 is a strain heterologous to the H1 FL HA A/Brisbane/59/2007 (87% HA amino acid (aa) identity) and H5N1 A/HongKong/156/97 is a strain genetically more distant and heterosubtypic to the H1 FL HA used for immunization (63% HA aa identity to H1 FL HA A/Brisbane/59/2007). The highest dose of 1030 nmol was included only in the H5N1 study to ascertain protection induced by vaccination could be observed against this genetically distant virus strain. The vaccine antigens induced comparable titers against H1 FL HA A/Brisbane/59/07 in both studies (Supplementary Figure 1), indicating similar immunogenicity across the studies.

**Figure 1:**
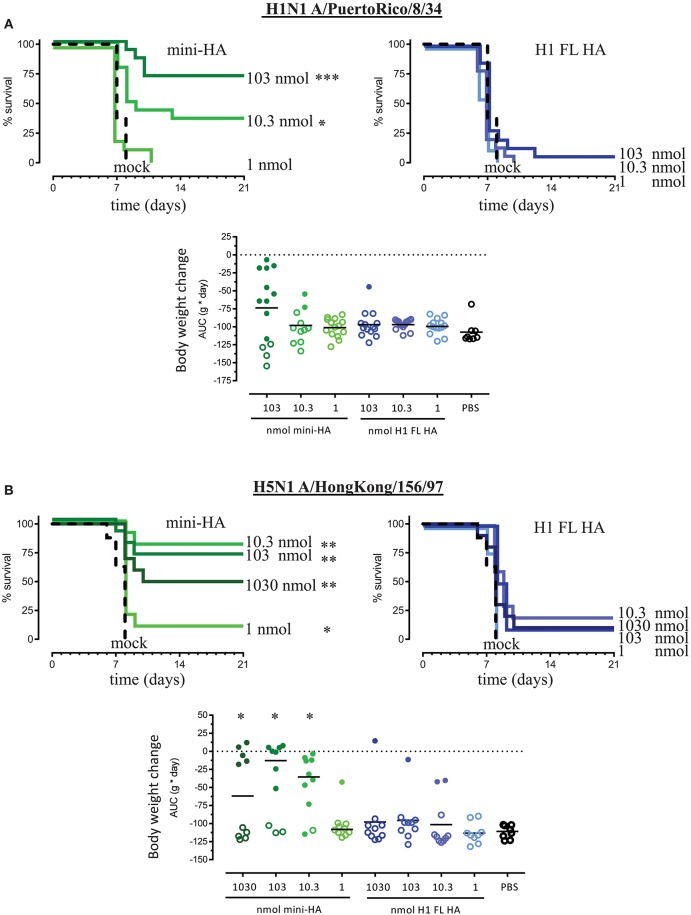
Immunization with mini-HA but not H1 FL HA induces cross-protection against H5N1 and H1N1. Mice received 103 nmol (3 μg mini-HA or 6 μg H1 FL HA), 10.3 nmol (0.3 μg mini-HA or 0.6 μg H1 FL HA) or 1 nmol (0.03 μg mini-HA or 0.06 μg H1 FL HA) of the antigens. Four weeks after the third immunization mice were challenged with 12.5LD_50_ of either influenza H1N1 A/PuertoRico/8/34 (**A**, 14 mice per group) or H5N1 A/HongKong/156/97 (**B**, 10 mice per group) and monitored 21 days for survival, body weight and median clinical score. 1,030 nmol (30 μg mini-HA or 60 μg H1 FL HA) vaccination was included only in the H5N1 challenge experiment. All mice in the mock control groups succumbed to the H1N1 and H5N1 challenge within 8 days. Upper graphs represent the Kaplan-Meier survival curves, lower graphs represent bodyweight change (gram x day) area under the curve (AUC). Solid lines indicate group medians, closed symbols indicated mice which survived subsequent influenza virus challenge. Asterisks indicate statistically significant differences in survival time or change in body weight compared to the mock immunized control group (^*^p < 0.05, ^**^p < 0.01, ^***^p < 0.001 according to the material and methods section).

H1 FL HA immunization did not induce significant protection against H1N1 A/PuertoRico/8/34 for any of the doses tested (Figure 1) compared to mock immunization. In contrast, immunization with 103 and 10.3 nmol of mini-HA significantly increased the duration of survival (*p* < 0.001 and *p* < 0.05, respectively) relative to mock immunization. When compared across all doses, mini-HA significantly increased the survival proportion (*p* < 0.001), duration (*p* < 0.001), and improved the clinical score (*p* < 0.001) against the lethal H1N1 challenge relative to immunization with H1 FL HA (Supplementary Figure 2A).

Immunization with H1 FL HA also did not induce significant protection against lethal H5N1 challenge at any dose (Figure 1B), compared to mock immunization, while mini-HA did induce significant protection against lethal heterosubtypic H5N1 A/Hong Kong/156/97 influenza challenge compared to mock immunization. When compared across all doses to H1 FL HA immunization, mini-HA significantly increased the survival proportion (*p* < 0.001) and duration (*p* < 0.001; Figure 1B), the clinical score (*p* < 0.001) and significantly reduced the body weight loss (*p* < 0.001; Supplementary Figure 2B) after lethal challenge with H5N1.

### Both mini-HA and H1 FL HA induce antibodies binding to a panel of group 1 FL HA

**Figure 2:**
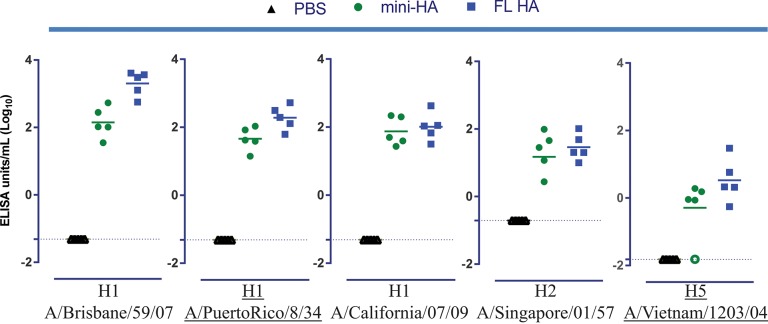
Similar titers of antibodies binding to a panel of group 1 FL HA induced by mini-HA and H1 FL HA immunization. Serum samples taken 4 weeks after the third immunization were analyzed for binding to a panel of group 1 influenza FL HA by ELISA. Results of mock immunization (PBS) or immunization with 103 nmol antigen are shown. 103 nmol results are representative for all doses tested, see Supplementary Figures 1, 3 for ELISA titers of all doses. Solid lines indicate the group means, dashed lines indicated the LOD. Open symbols represent samples at LOD. All serum samples taken from mock immunized mice are on LOD. Underlined influenza strains represent the FL HA homologous to, or the same subtype of, the challenge strains, see materials and methods.

To determine whether the cross-protection observed after mini-HA immunization is reflected by broadly reactive antibodies, we analyzed serum samples taken after the third vaccination, 1 day prior to challenge, for IgG antibody binding titers to a panel of influenza A group 1 recombinant FL HAs, see Figure 2. Immunization with 103 nmol mini-HA compared to 103 nmol H1 FL HA show similar results and are representative of the other doses tested, see Supplementary Figures 1, 3.

As expected, due to the presence of HA head epitopes, FL HA immunization compared to the mini-HA induces significant higher antibody titers specific for H1 FL HA A/Brisbane/59/07 (*p* < 0.001, comparison made across doses), the influenza strain homologous to the H1 FL HA vaccine strain, see Figure 2.

Unexpectedly, both the mini-HA and H1 FL HA immunization induced similar antibody titers against the other, phylogenetically more distant, FL HAs tested. This included antibody titers specific for H1 FL HA A/Puerto Rico/8/34 and H5 FL HA A/Vietnam/1203/04 which are homologous to the challenge models used, in clear contrast with the observed differences in cross-protection.

### *In vitro* cross-neutralization detected in IgG concentrated samples

Overall binding levels may obscure the fact that the polyclonal repertoires elicited by mini-HA and H1 FL HA immunization will likely bind to different neutralizing and non-neutralizing epitopes on FL HA. Therefore, we analyzed whether the observed difference in survival between immunization with mini-HA and H1 FL HA, despite similar FL HA antibody binding, could be explained by differences in neutralization.

**Figure 3:**
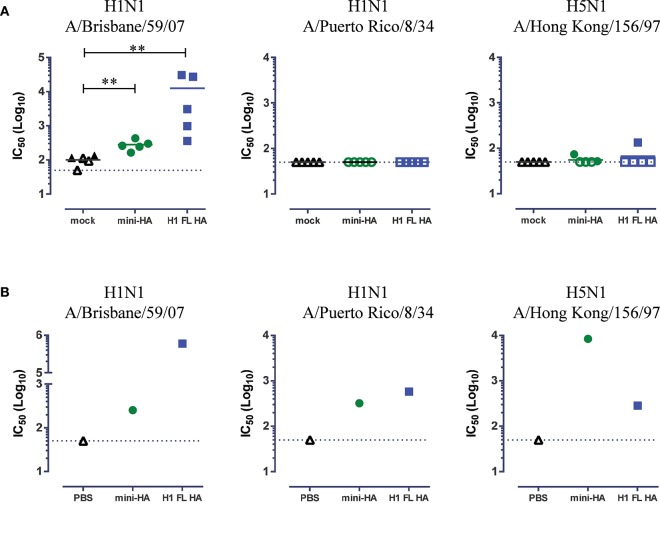
Cross-neutralization detected in concentrated serum samples. Neutralization of H1N1 A/Brisbane/59/07, H1N1 A/PuertoRico/8/34 and H5N1 A/HongKong/156/97 as measured by pseudoparticle VNA assay. Results of immunization with 103 nmol antigen shown. Serum samples were taken 1 day prior to influenza challenge. Results are shown for individual mice **(A)**, or mouse samples were pooled per immunization regimen and antibodies were purified and concentrated **(B)**. Solid lines indicate the group means. Closed symbols represent positive values, see material and methods. Dashed lines indicate LOD. Asterisks indicate statistical significant differences between regimens (^**^p < 0.01). No statistical test was done on the single replicates in **(B)**.

We tested serum samples taken from mice immunized with 103 nmol antigen, the highest common dose included in both challenge studies, 1 day prior to challenge for *in vitro* neutralization of influenza strains (Figure 3). Detectable neutralization titers of H1N1 A/Brisbane/59/07 were induced by both H1 FL HA and mini-HA immunization (Figure 3A, *p* < 0.01). Low, or no neutralization of the challenge strains was detected for H5N1 A/Hong Kong/156/97 and H1N1 A/Puerto Rico/8/34 respectively. However, as it is known that low levels of neutralizing antibodies can be relevant to protection *in vivo* (27), we repeated the neutralization assays after purification and concentration of antibodies of the pooled serum samples. We found detectable neutralization of H1N1 A/Brisbane/59/07 for both these concentrated samples of mini-HA and H1 FL HA induced antibodies, see Figure 3B. In addition, we detected neutralization of H1N1 A/Puerto Rico/8/34 and H5N1 A/Hong Kong/156/97. Neutralization titers induced by mini-HA and H1 FL HA immunization were similar against the H1N1 A/PuertoRico/8/34 strain. Corresponding with the H5N1 challenge results, there appears to be a trend that mini-HA induces higher H5N1 A/Hong Kong/156/97 neutralization titers than H1 FL HA, which could at least in part explain the differences seen in survival in this challenge model.

### Antibody dependent cell-mediated immunity

**Figure 4:**
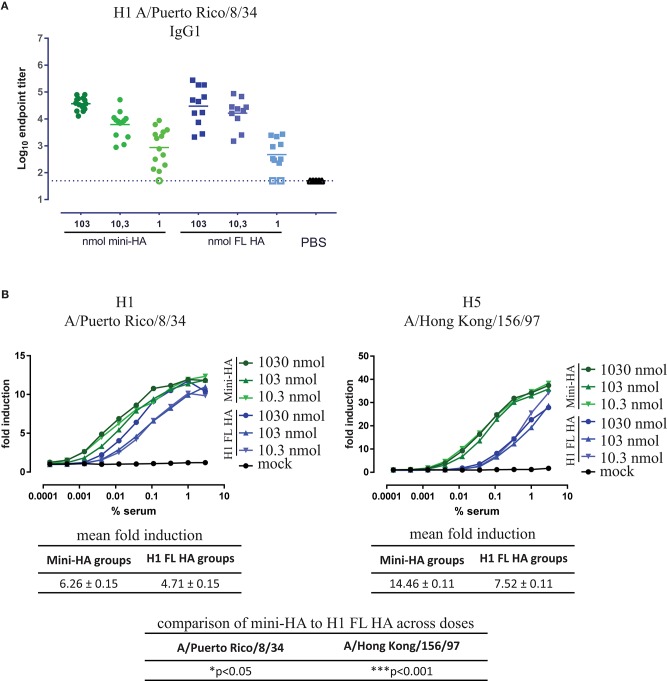
Similar IgG1 titers found but different mFcγRIII activation. Serum taken 1 day prior to influenza challenge were tested in ELISA for IgG1 binding to H1 FL HA A/Puerto Rico/8/34 **(A)** and for FcγRIII activation by H1 A/Puerto Rico/8/34 and H5 A/Hong Kong/156/97 in a surrogate ADCC assay **(B)**. Mouse samples were pooled per dose and immunization regimen for the ADCC assay **(B)**. Open symbols represent values at or below LOD. All serum samples taken from mock immunized mice (PBS) are on LOD. Solid lines indicate the group means, error bars are not depicted for better data visualization, dashed lines indicated the LOD. Mean fold induction tables show the means across doses of the mean fold induction per vaccination and replicate (indicated as mean fold induction in brief, also known as least square mean) ± the standard error of the mean. The mean fold induction of mini-HA was compared to the mean fold induction of H1 FL HA; ^*^p < 0.05, ^***^p < 0.001.

In addition to direct neutralization of virus, antibodies can contribute to protection by cytotoxicity to infected cells via interaction between the Fc region of the antibody and its receptors (FcγR) found on most immune cells. In mice, these effector functions such as antibody-dependent cellular cytotoxicity (ADCC) and antibody-dependent cellular phagocytosis (ADCP) are mainly mediated by IgG2a isotype antibodies activating mFcγRIV (28). However, overall HA A/Brisbane/59/07-specific IgG2a titers were low, due to the use of Alum, a known Th2 and subsequent IgG1 skewing adjuvant in BALB/c mice (29) (Supplementary Figure 4A). Subsequently, no mFcγRIV activation could be detected in a reporter assay (Supplementary Figure 4B). In contrast, HA A/Brisbane/59/07-specific IgG1 titers could be detected in all mice, except in those immunized with the lowest dose of H1 FL HA (Supplementary Figure 4A). However, again no difference between immunizations was found in IgG1 antibodies binding to H1 A/Puerto Rico/8/34 (Figure 4A), mirroring the total IgG response (Figure 2) and which again is not in line with the difference in protection. When tested for activation of mFcγRIII, the only activating Fcγ receptor in mice highly affine for murine IgG1, we found that immunization with mini-HA induced significantly higher Fcγ activation than H1 FL HA immunization against both H1 A/Puerto Rico/8/34 (*p* < 0.05) and H5 A/Hong Kong/156/97 (*p* < 0.001; Figure 4B), congruent with the challenge results. The disparity between a similar induction of IgG1 titers but different mFcγRIII activation between the immunization regimens could be explained by an inability of anti-HA head antibodies to engage cell-mediated effector functions through FcγRs, whereas HA stem-specific antibodies are largely dependent on this interaction (30, 31). To confirm this hypothesis, we looked into the HA target region of the antibodies induced by the immunization regimens, in particular to the receptor binding site (RBS) at the head in contrast to the HA stem region.

### Mini-HA induces more HA stem-binding antibodies, correlating to survival

**Figure 5:**
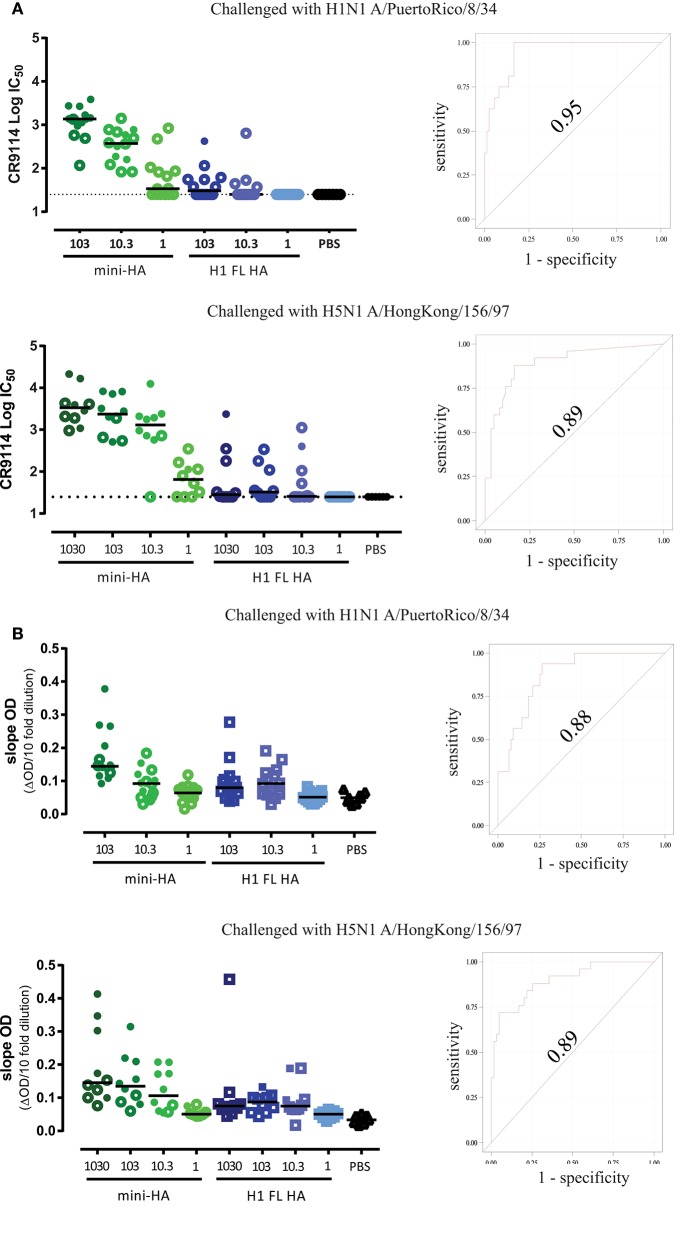
**(A)**. bnAb CR9114 competing antibody titers correlate with survival group 1 mini-HA used as antigen. Serum taken one day prior to challenge was tested for competition with bnAb CR9114, using mini-HA UFV4900 as antigen (left). Open symbols represent mice which did not survive subsequent challenge. Solid lines indicate the group medians. ROC correlation analyses of competition titers to challenge survival (right, see material and methods). Dashed lines indicate LOD. **(B)**. mCR9114 competing antibody titers correlate with survival H1 FL HA A/Puerto Rico/8/34 used as antigen. Serum taken one day prior to challenge was tested for competition with bnAb mCR9114 (a chimeric monoclonal antibody consisting of the variable domains of human CR9114 with a mouse IgG2a Fc constant domain), using H1 FL HA A/Puerto Rico/8/34 as antigen (left panels). Open symbols represent mice which did not survive subsequent challenge. Solid lines indicate the group medians. ROC correlation analyses of competition titers to challenge survival (right panels, material and methods).

As expected, H1 FL HA immunization induced antibodies blocking the RBS as measured by hemagglutination inhibition (HI) titers against the H1N1 A/Brisbane/59/07 used for immunization (Supplementary Figure 5). Homologous HI titers induced by the H1 FL HA are in line with the observed neutralization, as is the absence of detectable HI titers against heterologous H1N1 (Supplementary Figure 5). As expected, no HI titers were induced by mini-HA immunization against any of the strains tested. To determine whether mini-HA vaccination induces more HA stem-specific antibodies compared to H1 FL HA, we tested sera taken one day prior to challenge for competition with HA stem-specific broadly neutralizing antibody (bnAb) CR9114 (32). We found that mice immunized with the mini-HA, but not H1 FL HA, potently induced antibodies competing with CR9114 over binding to the influenza A group 1 stem (Figure 5A). Moreover, we found that these competition titers in individual mice were strongly correlated with protection observed after lethal H1N1 A/PuertoRico/8/34 or H5N1 A/HongKong/156/97 challenge (an area under the curve (AUC) of the Receiver operating characteristic (ROC) of 0.95 for H1N1 and 0.89 for H5N1). Similar results and correlations were found when H1 FL HA A/PuertoRico/8/34 was used as target antigen to measure CR9114 competition (AUC ROC of 0.88 for H1N1 challenge and 0.89 with H5N1 challenge; Figure 5B). Congruent with the competition assay, mini-HA induced higher antibody binding titers to the HA stem immunogen (UFV4900), than H1 FL HA immunization as analyzed by ELISA, see Supplementary Figure 6.

## Discussion

A promising new strategy in the development of broadly protective influenza vaccines is to direct the immune response toward the highly conserved HA stem region. By removing the immunodominant HA head a new so-called “mini-HA” was created and shown to induce cross-reactive and cross-protective antibodies in several animal models (22, 23, 26). However, the fact that a cross-protective immune response can also be observed in mice after multiple immunizations with a seasonal vaccine has raised the question whether in similar conditions, the mini-HA would show superiority over immunization with a H1 FL HA. Here we show in mice that in direct comparison to H1 FL HA, using the same regimen, dosing and adjuvant, mini-HA has a higher protective efficacy against group 1 influenza virus challenges not homologous to the H1 FL HA.

Cross-protection against influenza induced by immunization with the mini-HA or a seasonal vaccine previously has shown to be mediated by the humoral immune response in various studies (10, 23, 24, 26). This has been confirmed in our experiments by the absence of any detectable HA-specific T-cell responses (data not shown). However, the superior cross-protection induced by mini-HA compared to H1 FL HA immunization in our experiments did not translate into a difference in antibody binding titers to the FL HAs closely related to the challenge strains. Further analysis showed that in contrast to the similar overall binding levels, the antibodies elicited by the different immunization regiments bind to markedly different regions on the HA protein. In particular, even though both antigens contain the stem region of the HA protein, immunization with the H1 FL HA induced significantly less antibodies binding to the stem region. Confirming other studies comparing a HA-stem immunogen to FL HA immunization (18, 19, 22, 23, 26), our results show that the mini-HA overcomes these problems caused by the immuno-dominance of the HA head region, and elicits relatively high levels of stem-specific antibodies. We observed a markedly higher ratio between CR9114 competition titers and HA-stem binding antibody titers for mini-HA, relative to H1 FL HA immunization. This suggests that in addition to higher stem-specific antibody titers, mini-HA induced HA-stem binding antibodies could have a higher affinity, though this was not formally tested. When subsequently challenged, we found that the level of stem-specific antibodies induced by either H1 FL HA or mini-HA immunization strongly correlates to protection against lethal H1N1 and H5N1 influenza challenge. In human participants of a controlled pandemic 2009 H1N1 challenge trial, levels of HA-stem antibodies correlate with absence of influenza but not with disease severity in subjects which developed influenza (33). Another study found a correlation between the presence of HA stem-specific antibodies induced in humans and protection in mice after serum transfer (34). While the exact mechanism of protection by HA stem-binding antibodies cannot be deduced from the current experiments, HA stem-binding antibodies at least appear to serve as a surrogate marker of protection.

The difference in target location of the antibodies could explain an underlying difference in mechanism of protection mediated by HA head compared to HA stem-specific antibodies. Currently the only established correlate of protection by influenza vaccines is blocking of the receptor binding site on the HA head, as measured by the HI assay, thereby preventing attachment to the host cell. Antibodies specifically targeting the HA stem region in contrast neutralize influenza viruses by different mechanisms not detected by HI (35). There are various mechanisms by which anti-HA antibodies can directly neutralize influenza viruses such as by inhibition of membrane fusion, inhibition of HA0 cleavage and inhibition of egress. *In vitro*, we found that purified and concentrated antibodies from sera of mini-HA immunized mice can directly neutralize H5N1. The observed neutralization titers after immunization with mini-HA appeared to be substantially higher compared to immunization with H1 FL HA, which could at least in part explain the better protection observed against H5N1 influenza challenge. The better protective efficacy against lethal H1N1 influenza challenge however could not be explained by a difference in *in vitro* H1N1 neutralization. These results are in line with a recent study, where HA stem-specific antibody levels in humans were better predictors of challenge outcome in mice after serum transfer than neutralizing antibody levels (34).

In addition to direct neutralization, indirect mechanisms of action, mediated by immune cells or complement factors, might also play a role in protection. There is an increasing body of evidence showing FcγR-mediated ADCC and ADCP play important roles in protection mediated by stem-specific and non-neutralizing HA antibodies (30, 34, 36). The potency of an antibody to engage these effector functions could thus explain the better cross-protection induced by mini-HA immunization, in spite of similar HA-specific antibody titers. In mice these FcγR mediated effector functions are mediated mainly by IgG2a antibodies, binding with higher affinity to mFcγRIV and typically inducing a type 1 immune response. However, alum based adjuvants, as used in this study, typically induce more of a type 2 immune response reflected by predominantly IgG1 antibodies in mice (29). Therefore, as expected, even though HA-specific IgG2a antibodies could be detected after both mini-HA and H1 FL HA immunization, these levels were low and subsequently no mFcγRIV activation could be detected in an ADCC reporter assay which could explain the difference in protection.

In contrast to IgG2a which binds to several activating Fcγ receptors, including mFcγRIV, the only activating Fcγ receptor to which IgG1 binds is mFcγRIII (37). Despite the pivotal role of mFcγRIV in HA stem-specific antibodies mediating protection against influenza (30), there might also be a role for mFcγRIII as IgG1 isotype antibodies were shown to mediate partial protection against influenza through mFcγRIII, in absence of mFcγRIV activation (38). Similar to mFcγRIV, this receptor can be found on most effector cells in mice involved in cytotoxic, neutralizing and agonistic antibody activities. However, the binding affinity of IgG2a to mFcγRIV is higher than that of IgG1 to mFcγRIII (37). In addition, the mFcγRIII is often co-expressed on murine immune cells with mFcγRIIb, an inhibiting receptor which binds IgG1 with higher affinity than mFcγRIII, which might result in an overall inhibitory signal. However, relative expression levels of mFcγRIIb and mFcγRIII may vary, as well as distribution, making it difficult to predict the contribution of co-expressing cells to protection against influenza. For example, murine Natural Killer (NK) cells only express the mFcγRIII and have shown to play a role in ADCC mediated killing of influenza infected cells (39–41). Together these results suggest that additional Fc-mechanisms of protection correlating with HA stem-specific antibodies may play a role against influenza infection. Future studies, potentially making use of specific knock-out mouse models or adoptive transfer of specific immunological subsets, will have to show which immune cell populations contribute to protection. In conclusion, for the first time in a direct, well controlled, comparison to H1 FL HA immunization we show here that mini-HA induces higher levels of cross-protection in H1N1 and H5N1 challenge models, warranting further development of a universal influenza vaccine strategy based on the HA stem region. Although future studies will have to further address the mechanisms by which these stem-specific antibodies protect against influenza *in vivo*, collectively our results suggest that at least in mice several mechanisms, such as neutralization and cell mediated effector functions play a role.

## Materials and methods

### Statement of ethics

All mouse experiments were performed in accordance with Dutch legislation on animal experiments and approved by the DEC Consult, an independent ethical institutional review board.

### Immunization

Six week old female BALB/c mice (specific pathogen-free) were purchased from Charles River laboratories (Sulzfeld, Germany). The group 1 mini-HA UFV4900 (23) and H1 FL HA A/Brisbane/59/07 were produced in HEK293F cells (in-house). Using differential scanning fluorimetry, the mini-HA starts to unfold around 52°C, and H1 FL HA around 50°C, suggesting the thermal stability of the antigens to be similar. Fifteen mice per group (H5N1 challenge) or 14 mice per group (H1N1 challenge) were immunized three times via the intramuscular (i.m.) route 3 weeks apart with 100 μl vaccine (50 μl per hind leg). Mice received 1,030 nmol (30 μg mini-HA or 60 μg H1 FL HA), 103 nmol (3 μg mini-HA or 6 μg H1 FL HA), 10.3 nmol (0.3 μg mini-HA or 0.6 μg H1 FL HA) or 1 nmol (0.03 μg mini-HA or 0.06 μg H1 FL HA) of the antigens. Control groups received 100 μl PBS. All immunizations were adjuvanted with Al(OH)_3_, Alum (2% Alhydrogel®, Brenntag). Four weeks after the final immunization 5 mice per group of the H5N1 challenge study were euthanized and serum and spleens were collected to assess humoral and cellular responses, respectively. Four weeks after the final immunization, 1 day prior to challenge, mice in the positive challenge control group received bnAb CR6261 and subsequently a pre-challenge blood sample was obtained of all remaining mice via submandibular bleeding.

### Challenge

Four weeks after the final immunization the remaining mice (10 mice per group in the H5N1 challenge experiment, 14 mice per group in the H1N1 challenge experiment) were anesthetized by intraperitoneal (i.p.) administration of 100 mg/kg ketamine in combination with 20 mg/kg xylazine. Mice were challenged with 12.5xLD_50_ of H1N1 A/Puerto Rico/8/34 (performed at Janssen Vaccines & Prevention B.V., Leiden, the Netherlands) or 12.5xLD_50_ H5N1 A/Hong Kong/156/97 (performed at CVI Lelystad, the Netherlands) via the intranasal route (a total of 50 μl, 25 μl per nostril). Bodyweight and clinical score were monitored daily for up to 21 days or until the humane endpoint to limit animal discomfort. Humane endpoint was defined based on clinical score as is established practice (32, 42–44). All mice in the PBS control groups succumbed to the H1N1 and H5N1 challenge within 8 days. All mice in the bnAb CR6261 control groups survived the H1N1 and H5N1 challenge.

### ELISA

To assess HA-specific antibody binding levels in serum samples, recombinant FL HA of H1 A/California/07/09, H1 A/Puerto Rico/8/34, H1 A/Brisbane/59/07, H2 A/Singapore/01/57 or H5 A/Vietnam/1203/04 (94% sequence identity to H5 A/Hong Kong/156/97 were obtained from Protein Sciences Inc., CT, USA and produced on *expres*SF+ insect cells) were coated at a concentration of 0.5 μg/mL onto Maxisorp 96-well plates (Nunc, Thermo Scientific) O/N at 4°C. Plates were washed with PBS (Life Technologies, Paisley, UK) containing 0.05% Tween-20 (Merck Millipore, Darmstadt, Germany) (PBS-T) and subsequently blocked with PBS containing 2% dried skimmed milk (BD, Breda, the Netherlands) for 1 h at RT. Following a wash with PBS-T, serum of individual mice were added to the plate in duplicate, serially diluted (2-fold, 0.002–2%) and incubated for 1 h at RT. Following a wash with PBS-T a 1:2,000 dilution of goat-anti-mouse Horse Radish Peroxidase (HRP) conjugated (KPL, Maryland, USA) was added to the plate and incubated for 1 h at RT. After washing with PBS-T, o-phenylenediamine dihydrochloride (OPD) substrate (Thermo Scientific) was added to the plate. The colorimetric reaction was stopped after 10 min by adding 1 M H_2_SO_4_. The optical density (OD) was measured at 492 nm and standard curves were created using a four-parameter logistic curve. The OD of each sample dilution was then quantified against the standard mCR9114 (a chimeric monoclonal antibody consisting of the variable domains of human CR9114 with a mouse IgG2a Fc constant domain, produced in-house) for all FL HA except H2 A/Singapore/01/57, for which murine C179 (Takara) was used as standard) and the final concentration per sample (in ELISA Units, EU/ml) calculated by a weighted average, using the squared slope of the standard curve at the location of each quantification as weight. Negative samples were set at the limit of detection (LOD), defined as the lowest sample dilution multiplied by the lowest standard concentration with an OD response above the lower asymptote of the standard curve or background, whichever was higher.

IgG isotype ELISAs were performed similarly, except using a 1:1,000 dilution of HRP-conjugated IgG1 and IgG2a (Southern Biologics, Florida, USA) as detection antibody. The optical density (OD) was measured at 492 nm and curves were created using a four-parameter logistic curve. The ELISA endpoint titers were calculated as the reciprocal of the dilution of test sera that gave an *OD* >4 times the mean of all negative controls per plate (8 wells without serum).

### Pseudoparticle neutralization assay

Pseudoparticle neutralization assays were performed by Monogram Biosciences. Pseudoviruses expressing HA were generated. The pseudovirus stocks, at a concentration giving approximately 30,000–300,000 relative light units (RLU) per well, were incubated at 37°C for 1 h with 3 or 4-fold serial dilutions of test sera in a 96 well plate starting at a 1:10 dilution or higher. HEK293 cells were then added to each well and incubated at 37°C in 5% CO_2_ for 3 days. Luciferase substrate and cell lysing reagents were added to the plates which were read on a luminometer. Data analysis was performed by Monogram Biosciences. Inhibition curves were defined by a four-parameter sigmoidal function and were fit to the data by nonlinear least-squares regression and bootstrapping. These were used to calculate the antibody/drug concentration required to inhibit virus infection by 50% (ID_50_/IC_50_). The ability of antibody in the serum to neutralize influenza infectivity was assessed by measuring luciferase activity in the culture 72 h after viral inoculation as compared to a control infection using a murine leukemia virus envelope (aMLV-env) pseudotyped virus. Neutralization titers are expressed as the reciprocal of the serum dilution that inhibited the virus infection by 50%. A sample was called positive for neutralization when there was at least 50% inhibition of infection of an influenza virus strain and when there was an IC_50_ at least 3-fold higher than the IC_50_, if any, of the same sample tested with the specificity control, aMLV-env.

To increase sensitivity of the assay, serum samples were pooled per immunization regimen and IgG antibodies were purified and concentrated approximately a factor 30 by volume. Protein A/G Beads (Genscript) were vortexed and washed three times before use according to manufacturers' instructions. Beads were resuspended in binding/washing buffer (20 mM Na_2_HPO_4_, 0.15 M NaCl, pH 7.0). Per sample, 1:25 dilution of serum was added to the beads, and mixed by gently inverting the tube, and incubated at RT on a roller shaker for 30 min. The tubes were placed in a magnetic rack and supernatant was discarded. Samples were washed three times with binding/washing buffer. Three times elution buffer (0.1 M glycine, pH 2–3) was added, mixed, and incubate for 5 min at RT on a roller shaker. The tubes were placed in a magnetic rack and supernatant was collected in neutralization buffer (1 M Tris, pH 8.5). To remove the residual beads, tubes with eluted IgG were place in a separation rack and eluted IgG were transferred to a fresh tube. Samples were concentrated with Amicon Ultra-0.5 centrifugal filter device (50 K, EMD Millipore, Massachusetts, United States) according to manufacturers' instructions. Briefly, samples were spun 14,000 g for 10 min multiple times and resuspended in antibody buffer (DPBS, Gibco). Samples were spun again at 14,000 g for 10 min until sample volumes were concentrated by approximately a factor 30 of starting sample volumes.

### ADCC reporter assay

Signaling by the mFcγRIII and IV activation as measured in the ADCC reporter assay was performed as previously described (23, 44). Briefly, human lung carcinoma–derived A549 epithelial cells were maintained in Dulbecco's modified eagle medium (DMEM) supplemented with 10% heat inactivated fetal calf serum (Gibco) at 37°C, 10% CO_2_. Two days before the experiment, A549 cells were transfected with plasmid DNA encoding for full length H1 A/Puerto Rico/8/34 or H5 A/Hong Kong/156/97 using Lipofectamine 2,000 (Invitrogen) in Opti-MEM (Modified Eagle Medium, Invitrogen). One day before the assay, transfected cells were harvested and seeded in white 96-well plates (Costar). After 24 h, samples were diluted in assay buffer (4% ultra-low IgG FBS (Gibco) in Roswell Park Memorial Institute medium (RPMI 1,640, Gibco) and heat inactivated for 30 min at 56°C, followed by serial dilution in assay buffer. After washing with PBS the cells were replenished with fresh assay buffer and antibody dilutions and ADCC Bioassay effector cells (a stable Jurkat cell expressing mFcγRIII or mFcγRIV, human CD3γ, and an NFAT response element regulating a luciferase reporter gene), were added and incubated for 6 h at 37°C at a target-effector ratio of 1 to 3 (mFcγRIII) or 1 to 4.5 (mFcγRIV). Cells were equilibrated to room temperature for 15 min before Bio-Glo Luciferase System substrate (Promega, Madison, US) was added. Luminescence was read out after 10 min on a Synergy Neo (Biotek, Winooski, US). BnAb mCR9114 (described above) was used as a positive control for both reporter cells. Data are expressed as mean fold change of signal in the absence of serum over all concentrations and replicates.

### Competition assay CR9114

Antibodies competing with CR9114 over a broadly neutralizing epitope on the HA stem were determined as previously described (23). Briefly, Maxisorp 96-well plates (NUNC) were coated o/n with purified polyclonal rabbit anti His-Tag IgG (Genscript USA Inc., NJ, US) followed by washing with PBST. After blocking with PBS/ 2% BSA and washing, plates were incubated with His-tagged mini-HA UFV4900 or H1 FL HA A/Puerto Rico/8/34 for 2 h at RT. Plates were washed and serum added to the plate in duplicate, serially diluted in block buffer and incubated for 1 h at RT. Subsequently a titrated amount of biotinylated human CR9114 was added and incubated for 1 h at RT. After washing streptavidin-HRP was added and incubated for 1 h at RT, followed by washing and OPD development. Positive controls consisted of competition with bnAb mCR9114 (as described above). OD was measured and fitted to a 4 parameter logistic curve. IC_50_ titers are expressed as the reciprocal of the serum dilution that inhibited bnAb mCR9114 binding by 50%. When S-curves were incomplete (as with the HA A/Puerto Rico/8/34 as target antigen), the CR9114 competition of each sample was quantified as the slope of the linear regression of OD value on the log 10 dilution for the duplicate series.

### HI

HI assay was performed as described (23). Shortly summarized, non-specific agglutination inhibitors were removed from serum samples incubation with *Vibrio cholerae* neuraminidase (Sigma-Aldrich) which was subsequently inactivated by incubation with 2.5% sodium citrate. Turkey red blood cells (Envigo) diluted in PBS were added, incubated and subsequently spun down. Twofold serial dilutions of the supernatant in PBS were prepared in duplicate, mixed by agitation with 4 HA units wild-type viruses H1N1 A/California/07/2009 or H1N1 A/Brisbane/59/07, and incubated followed by addition of Turkey red blood cells. Plates were again incubated and the hemagglutination status of each well was visually determined. The assay titer of a given serum sample was defined as the reciprocal of the highest dilution where no hemagglutination inhibition was observed.

### Statistical analyses

Statistical differences between immunization with mini-HA and H1 FL HA were evaluated for HA-specific binding antibodies, CR9114-competing antibodies, ADCC reporter assays and in the pseudoparticle neutralization assays. Data were log-transformed except for the ADCC assay. For the ADCC assay mean fold changes across antibody concentrations were calculated. Comparisons between the immunizations were made across doses using the Cochran-Mantel-Haenszel test, except for ADCC where an analysis-of-variance was used with dose and immunization as factors.

Survival proportion and survival time after challenge were analyzed using Fisher's-exact test and log-rank test, respectively, with Bonferroni adjustment for multiple comparisons. The survival proportions in the mini-HA groups were compared to the survival proportions in the H1 FL HA groups across doses with logistic regression assuming equally steep dose-response curves. The survival durations in the mini-HA groups were compared to the survival durations in the H1 FL HA groups across doses with proportional hazard regression assuming a constant hazard ratio between the immunization survival curves per dose. The differences in survival proportion and duration between the vaccines were tested with the corresponding *t*-test. Bodyweights and clinical scores were summarized as a single outcome per animal using an area under the curve (AUC) approach where missing values for animals that died early were imputed with a last-observation-carried-forward method. Bodyweight data are expressed as the change relative to the day 0 measurement. The AUC was defined as the summation of the area above and below the baseline. An ANOVA on AUCs was performed with group as explanatory factor. The AUC of clinical scores were analyzed by cumulative logistic regression with score level and with immunization as factors, log-transformed dose as covariable and mouse as subject. The differences between the vaccines were tested with the corresponding *z*-test.

Statistical analyses were performed using SAS version 9.4 (SAS Institute Inc. Cary, NC, US) and SPSS version 20 (SPSS Inc., IL, United States). Statistical tests were conducted two-sided at an overall significance level of α = 0.05.

## Data availability

The data that support the findings of this study are available from the corresponding author upon reasonable request, without undue reservation, to any qualified researcher.

## Author contributions

JvdL, WM, JT, RR, HK, and RZ: conceived and designed the experiments; JvdL, JH, JV, and SS-T: performed the experiments; JvdL, JT, and HK: analyzed the data; JvdL, HK, and LD: coordination animal studies; JvdL, RR, HK, RZ, TK, and BB: drafted the paper.

### Conflict of interest statement

The authors declare that the research was conducted in the absence of any commercial or financial relationships that could be construed as a potential conflict of interest. All authors are, or have been, employees of Janssen Vaccines & Prevention BV Pharmaceutical companies of Johnson and Johnson.
