# Supplementary material for: Mini-HA Is Superior to Full Length Hemagglutinin Immunization in Inducing Stem-Specific Antibodies and Protection Against Group 1 Influenza Virus Challenges in Mice
Source: Front Immunol. 2018 Oct 12;9:2350. doi: 10.3389/fimmu.2018.02350 (PMC6194913; doi:10.3389/fimmu.2018.02350)
Supplement: Supplementary file 1 [file Image_1.pdf]

Supplementary figures

1) Comparable immunogenicity of the antigens in both studies

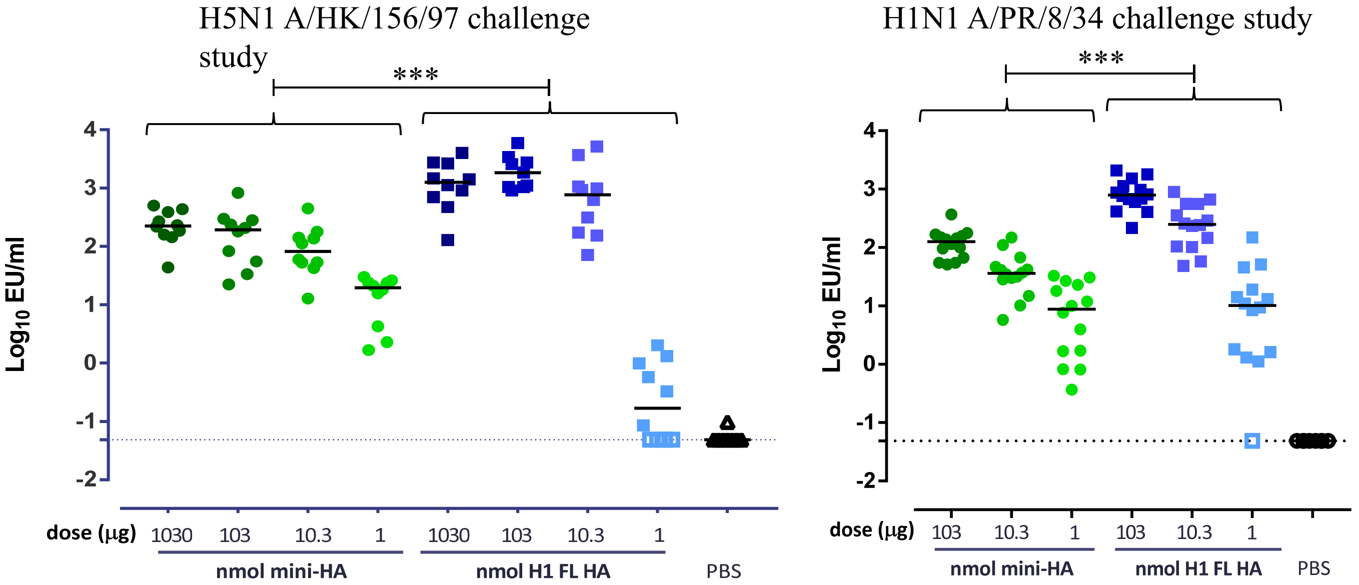

Serum samples taken one day prior to challenge were analysed for binding to H1 FL HA A/Brisbane/59/07. 103 nmol dose of the H5N1 A/HongKong/156/97 challenge experiment are shown in figure 2. Open symbols represent values at or below LOD. Solid lines indicate the group medians, dashed lines indicated the LOD. Asterisks indicate statistical significant difference across doses between mini-HA and H1 FL HA immunization (***$p<0.001$).

<u>**Supplementary figures**</u>

2) Body weight change, median clinical scores and statistical analyses lethal influenza challenges

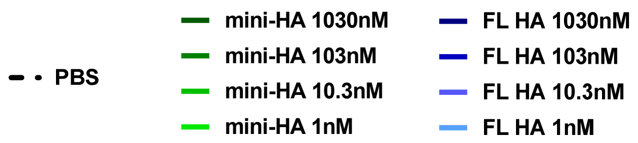

A

H1N1 A/PuertoRico/8/34

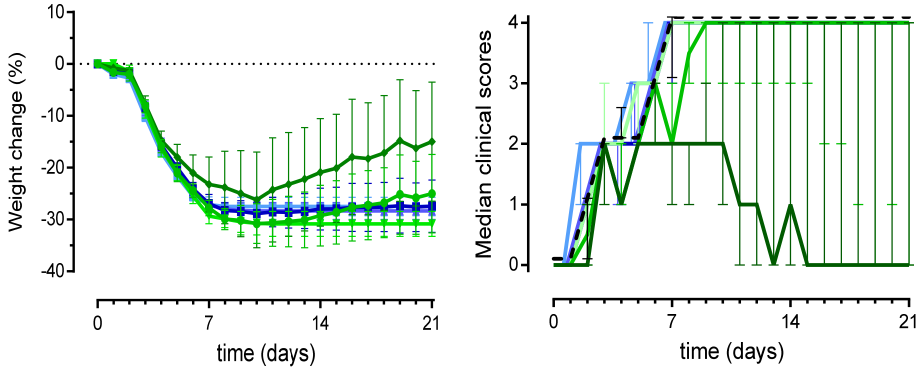

comparison of mini-HA over H1 FL HA across doses

Clinical score	Body weight loss
***p<0.001	p = 0.235

B

H5N1 A/HongKong/156/97

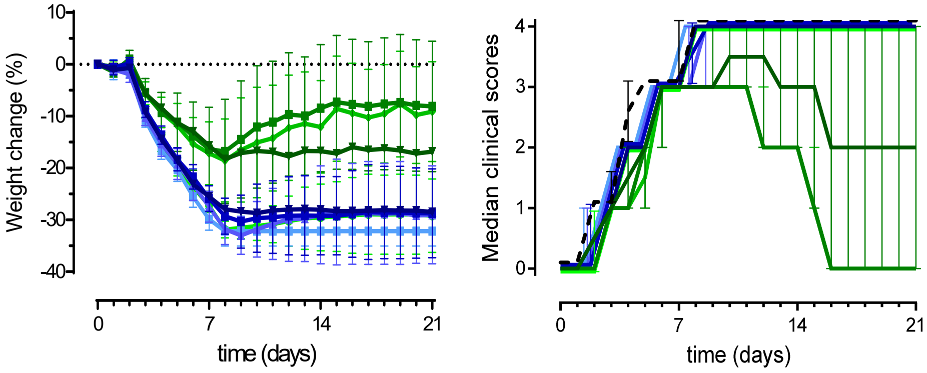

comparison of mini-HA over H1 FL HA across doses

Clinical score	Body weight loss
***p<0.001	***p<0.001

Four weeks after the third vaccination mice were challenged with 12.5LD_{50} of either influenza H1N1 A/PuertoRico/8/34 (panel A) or H5N1 A/HongKong/156/97 (panel B) and monitored 21 days for survival, body weight and median clinical scores. 1030nmol vaccination was included only in the H5N1 challenge experiment. Graphs represent the mean bodyweight change and median clinical scores, error bars denoting 95% confidence interval (body weight) or inter-quartile ranges (clinical scores). Tables show the statistical comparisons between mini-HA and H1 FL HA immunization across doses (***p<0.001 according to the material and methods section).

Supplementary figures

3) Antibodies binding to a panel of group 1 FL HA induced by mini-HA and FL HA immunization, all doses.

H1 A/PuertoRico/8/34

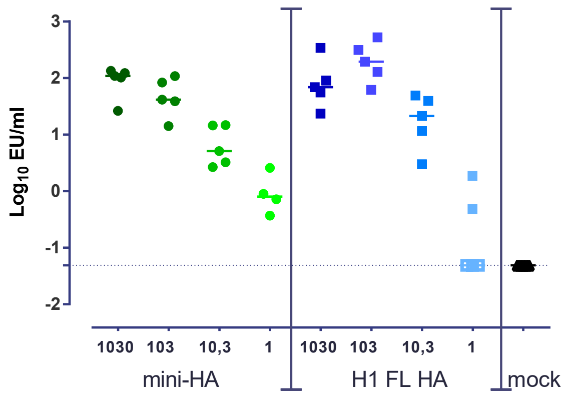

H1 A/California/07/09

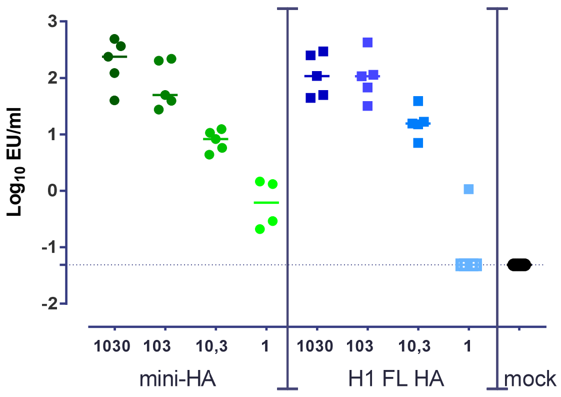

H2 A/Singapore/01/57

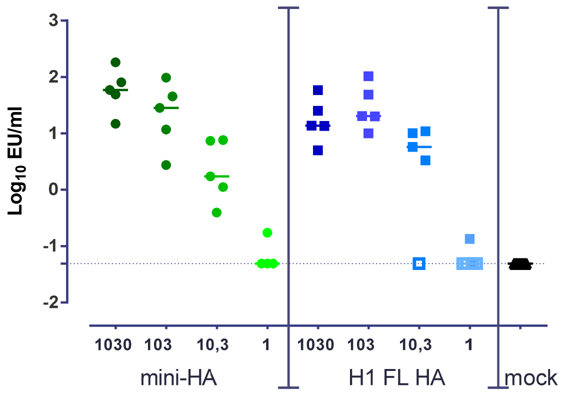

H5 A/Vietnam/1203/04

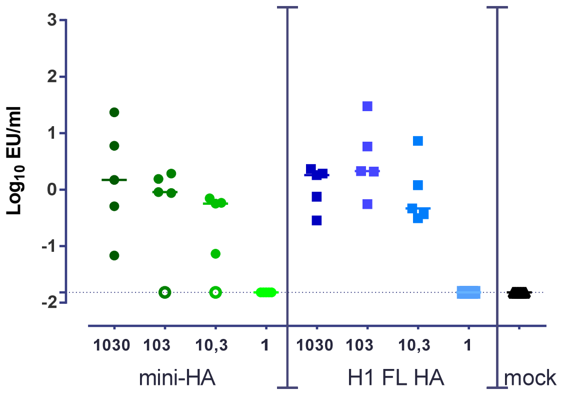

Serum samples taken one day prior to challenge were analysed for binding to a panel of group 1 influenza FL HA by ELISA. Open symbols represent values at or below LOD. All serum samples taken from mock immunized mice are on LOD. Solid lines indicate the group medians, dashed lines indicated the LOD. Underlined influenza strains represent the FL HA homologous or same subtype to the challenge strains, see materials and methods.

Supplementary figures

4) Low IgG2a and no FcγRIV response by Alum adjuvanted immunization

A

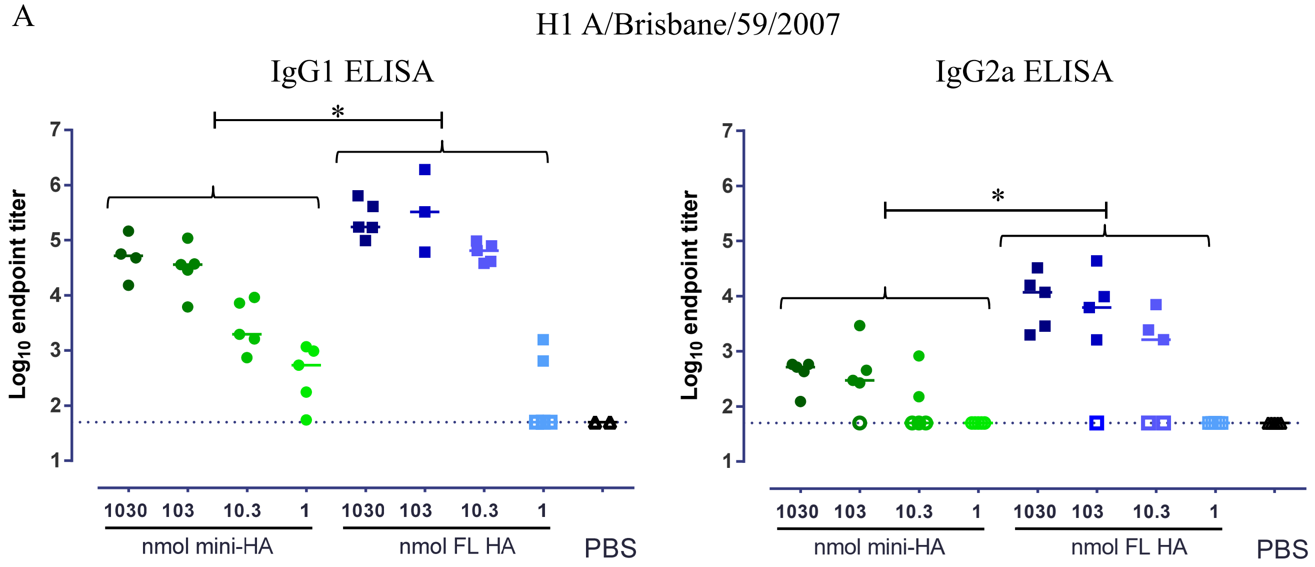

B

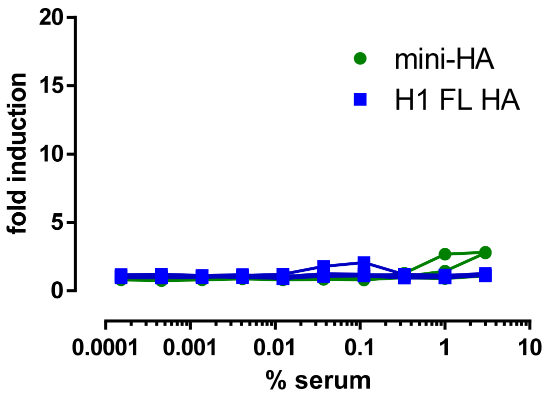

Serum samples taken one day prior to challenge were analysed for IgG1 and IgG2a binding to H1 FL HA A/Brisbane/59/07 (panel A), and for FcγRIV activation by H1 A/Brisbane/59/07 (panel B). A) Open symbols represent values at or below LOD. All serum samples taken from mock immunized mice (PBS) are on LOD. Closed lines indicate the group medians, dashed lines indicated the LOD. Asterisks indicated statistical significant differences between mini-HA and H1 FL HA immunization across doses, see materials and methods (*$p<0.05$). B) Serum samples of mice immunized with 1030nmol mini-HA or H1 FL HA were analyzed for FcγRIV activation by ADCC assay.

Supplementary figures

5) H1 FL HA immunization induces H1N1 A/Brisbane/59/07-specific HI titers

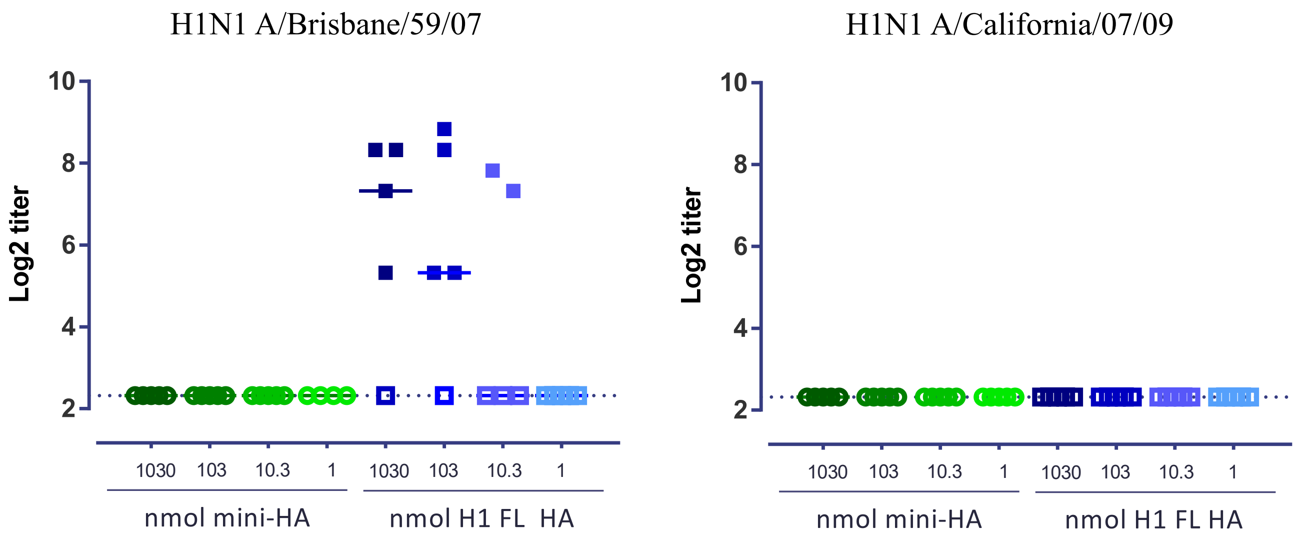

Serum samples taken one day prior to challenge were tested for inhibition of haemagglutination by H1N1 A/Brisbane/59/07 and H1N1 A/California/07/09. Solid lines indicate group medians, dashed lines indicates the LOD. Open symbols represent values at or below LOD.

Supplementary figures

6) Antibodies binding to the HA stem induced by mini-HA and FL HA immunization

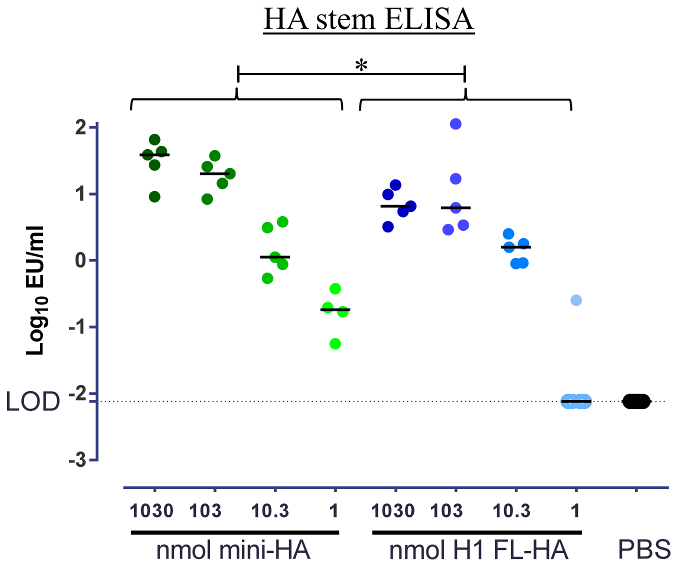

Serum samples taken one day prior to challenge were analysed for binding to the HA stem (mini-HA, UFV4900) by ELISA. Open symbols represent values at or below LOD. All serum samples taken from mock immunized mice are on LOD. Solid lines indicate the group medians, dashed lines indicated the LOD. Asterisk indicates statistical significant difference between mini-HA and H1 FL HA immunization across doses, see materials and methods (*$p<0.05$).
